# Comprehensive Evaluation of Advanced Imputation Methods for Proteomic Data Acquired via the Label-Free Approach

**DOI:** 10.3390/ijms252413491

**Published:** 2024-12-17

**Authors:** Grzegorz Wryk, Andrzej Gawor, Ewa Bulska

**Affiliations:** 1 Faculty of Physics, University of Warsaw, Pasteura 5, 02-093 Warsaw, Poland; wrykgrzegorz@gmail.com; 2 Biological and Chemical Research Centre, Faculty of Chemistry, University of Warsaw, Zwirki i Wigury 101, 02-089 Warsaw, Poland; ag.gawor@uw.edu.pl

**Keywords:** proteomics, label-free, data imputation, missing data, computational methods, advanced analytics, proteomic datasets, bioinformatics

## Abstract

Mass-spectrometry-based proteomics frequently utilizes label-free quantification strategies due to their cost-effectiveness, methodological simplicity, and capability to identify large numbers of proteins within a single analytical run. Despite these advantages, the prevalence of missing values (MV), which can impact up to 50% of the data matrix, poses a significant challenge by reducing the accuracy, reproducibility, and interpretability of the results. Consequently, effective handling of missing values is crucial for reliable quantitative analysis in proteomic studies. This study systematically evaluated the performance of selected imputation methods for addressing missing values in proteomic dataset. Two protein identification algorithms, FragPipe and MaxQuant, were employed to generate datasets, enabling an assessment of their influence on im-putation efficacy. Ten imputation methods, representing three methodological categories—single-value (LOD, ND, SampMin), local-similarity (kNN, LLS, RF), and global-similarity approaches (LSA, BPCA, PPCA, SVD)—were analyzed. The study also investigated the impact of data logarithmization on imputation performance. The evaluation process was conducted in two stages. First, performance metrics including normalized root mean square error (NRMSE) and the area under the receiver operating characteristic (ROC) curve (AUC) were applied to datasets with artificially introduced missing values. The datasets were designed to mimic varying MV rates (10%, 25%, 50%) and proportions of values missing not at random (MNAR) (0%, 20%, 40%, 80%, 100%). This step enabled the assessment of data characteristics on the relative effectiveness of the imputation methods. Second, the imputation strategies were applied to real proteomic datasets containing natural missing values, focusing on the true-positive (TP) classification of proteins to evaluate their practical utility. The findings highlight that local-similarity-based methods, particularly random forest (RF) and local least-squares (LLS), consistently exhibit robust performance across varying MV scenarios. Furthermore, data logarithmization significantly enhances the effectiveness of global-similarity methods, suggesting it as a beneficial preprocessing step prior to imputation. The study underscores the importance of tailoring imputation strategies to the specific characteristics of the data to maximize the reliability of label-free quantitative proteomics. Interestingly, while the choice of protein identification algorithm (FragPipe vs. MaxQuant) had minimal influence on the overall imputation error, differences in the number of proteins classified as true positives revealed more nuanced effects, emphasizing the interplay between imputation strategies and downstream analysis outcomes. These findings provide a comprehensive framework for improving the accuracy and reproducibility of proteomic analyses through an informed selection of imputation approaches.

## 1. Introduction

One of the most widely used approaches in mass spectrometry-based proteomics is the ‘label-free’ strategy. This method is favored for its ability to identify a vast number of proteins in a single analysis, making it particularly well-suited for large-scale proteomic studies and the discovery of novel biomarkers [1]. Unlike isotope labeling methods, which require additional sample preparation steps, the label-free approach is simpler and more cost-effective. Its versatility and high-throughput nature make it an indispensable tool in proteomic research, especially when dealing with complex biological samples or when a comprehensive overview of the proteome is needed. However, despite its advantages, the label-free strategy has some notable limitations. Compared to isotope labeling methods, it offers lower accuracy and reproducibility [2]. This is largely due to the inherent variability in the data acquisition process and the reliance on indirect measures of protein abundance, such as spectral counts or ion intensities. As a result, achieving consistent and reliable quantification across multiple samples can be challenging. One of the most significant obstacles in using the label-free strategy is the prevalence of missing values, which can affect up to 50% of the dataset [3]. These missing values, which often arise from technical and biological factors, represent a major barrier to robust data interpretation. They limit the ability to derive comprehensive and accurate insights into protein abundance, statistical relationships, and functional roles within the analyzed samples [4]. The incomplete data can skew results, reduce statistical power, and complicate downstream analyses, making it critical to address these gaps effectively. Thus, while the label-free strategy is a powerful tool, overcoming its limitations—particularly those related to missing values—is essential for maximizing its potential in proteomic research.

The causes of missing values are numerous and often challenging to pinpoint for any given protein. Missing values may arise from biological, instrumental, or sample preparation issues. Biological factors include the absence of a specific protein in the sample or its low concentration, which may fall below the detection limit of the instrument. Instrumental factors encompass issues such as ion competition, poor ionization efficiency, the selective fragmentation of only the most intense peptides during data-dependent acquisition (DDA), and errors in peptide identification [3]. Sample preparation may also contribute to missing values due to protein losses or irregular cleavages during trypsin digestion. To better understand missing values, they are commonly categorized as missing at random (MAR) or missing not at random (MNAR) [5]. The MAR values typically result from technical limitations and stochastic fluctuations, whereas MNAR values are directly correlated with the concentration of a specific protein or peptide in the sample [6]. Experimentally derived datasets often contain a mixture of both MAR and MNAR values. Although the precise ratio of MAR to MNAR values is generally unknown, it is widely believed that MNAR values constitute the majority [7]. Addressing missing values is crucial for ensuring the reliability of proteomic analyses. There are three main strategies to manage missing values. The first approach involves removing proteins that contain missing values in any sample. While straightforward, this method may result in the loss of valuable biological information. The second approach focuses on performing data analysis only on complete datasets with correctly identified values. However, this strategy significantly limits the use of many statistical methods, which require a complete dataset. The third and most commonly used approach is the imputation of missing values. This method replaces missing values with estimated values, allowing for the retention of valuable information and enabling robust data analysis while mitigating the impact of missing data on the overall dataset.

Removing all proteins that contain any missing values for some samples can result in the loss of valuable biological information. This approach may seem straightforward, but it often disregards the underlying complexity of biological systems, leading to the exclusion of relevant proteins that may still provide critical insights. On the other hand, performing data analysis solely on correctly identified values severely limits the applicability of most statistical methods, as these techniques typically require a complete dataset to generate reliable and meaningful results. The inherent challenges of missing values in proteomics datasets necessitate alternative approaches to handle this issue effectively. One widely adopted solution is data imputation, which relies on the assumption that all samples have fundamentally similar protein compositions, with observed differences reflecting variations in the expression levels of individual proteins. By replacing missing values with estimated values, imputation minimizes the loss of information and allows for more robust statistical analyses. In proteomic datasets, a left-censored normal distribution is often observed, resulting from the non-random nature of missing values, which are often missing not at random (MNAR) [3]. As a result, it is widely believed that imputation methods should be specifically designed to handle left-censored data, allowing for the effective approximation of small MNAR values [7]. However, imputation is not without its challenges. As the number of missing values increases, the potential for distortion in the data grows, leading to increasingly biased or inaccurate estimates [8,9]. For instance, assigning a constant value to a protein (such as the mean or median of non-missing values) can cause an underestimation of the variance, which in turn may result in false-positive findings. This highlights the importance of carefully selecting the most appropriate imputation strategy to avoid introducing systematic errors into the analysis.

Imputation methods are generally classified into three main types: single-value imputation, local-similarity methods, and global-similarity methods. Each of these approaches has its own strengths and limitations, depending on the nature of the dataset and the specific goals of the analysis. Single-value imputation methods, for example, are typically simpler to implement but may introduce significant bias, especially when missing values are not randomly distributed. In contrast, local-similarity methods are based on the assumption that proteins with similar expression patterns can be used to predict missing values, making them more suited to datasets where proteins show strong interdependencies. On the other hand, global-similarity methods adopt a broader approach by using overall patterns across the entire dataset to reconstruct missing data, which can be particularly useful when dealing with complex proteomic data. The choice of imputation method depends on both the characteristics of the data and the underlying assumptions about the causes of missing values.

Single-value imputation methods replace missing values with a constant or random value. This category includes LOD (imputation of lowest acquired intensity from entire dataset), SampMin (imputation of lowest acquired intensity for a given protein) and imputation of a random value from the left tail of the normal distribution (ND), also known as RTI (random tail imputation) [8].

Local-similarity imputation methods are based on the assumption that protein behaviors and expression are interdependent and highly correlated [9]. The fundamental idea behind these methods is to estimate missing values by leveraging the information from other proteins that exhibit similar intensity profiles. The process typically begins by identifying a set of proteins that closely resemble the imputed protein in terms of expression patterns. Then, the missing value is calculated as a weighted combination of the intensities of these similar proteins. Several algorithms fall under this category, including k-nearest neighbors (kNN), local least-squares (LLS), least-squares adaptive (LSA) and random forest (RF). Each of these methods utilizes different techniques to identify and combine information from similar proteins, allowing for more accurate imputation of missing data. Imputation methods based on global similarity use matrix reduction techniques to iteratively reconstruct missing values. Among such methods are SVD (single-value decomposition) and variations of the PCA method: PPCA (probabilistic principal component analysis), and BPCA (Bayesian principal component analysis).

At this moment, there is no consensus in the field of proteomics on the best method for data imputation. Problems that lead to this situation include the lack of comparable, high-quality datasets, the use of different imputation methods depending on the publication, and the selection of criteria for assessing imputation efficiency. A reasonable approach seems to be a direct comparison of experimentally obtained values with the values imputed by the chosen algorithm. A popular strategy is to simulate missing values on the full dataset [10,11]. This allows for the calculation of differences between real and imputed values, but studies on real missing values are still most desirable. This forces the definition of other evaluation criteria than a simple comparison, e.g., through statistical parameters [6].

The studies conducted [6,10,11,12,13,14] show that different imputation methods present different performances on MAR and MNAR values. Single-value imputation methods showed good performance on MNAR values in contrast to local-similarity methods, which performed well on MAR values. The opposite was the case for methods such as SVD, kNN, or REM [10]. The conclusions suggested by Kong et al. [11] indicate that the selection of the imputation method should depend on the characteristic and structure of the experimentally obtained data; while this is a very difficult task in itself, it is strongly desirable to find a universal way of proceeding and select the best imputation method regardless of the data. It is difficult to choose the best imputation method based on the available results. In the work carried out so far, the best methods have been identified as follows: SampMin [12,13,14], belonging to the single-value imputation group; REM, LSA [12], kNN [13,14,15,16], and RF [6,11,16,17,18,19,20] from the family of methods based on local similarities; and BPCA [19], based on global similarities.

The primary objective of this study is to systematically collect and evaluate imputation methods previously identified as highly effective in the scientific literature [6,10,11,12,13,14,15,16,17,18,19,20], applying them to a real proteomics dataset obtained through manual curation. The analysis encompasses widely utilized approaches for data handling and integrates representative methods from all major imputation categories: single-value, local-similarity, and global-similarity strategies.

As single-value methods, two commonly used left-censored approaches, LOD and ND, were selected, as well as the SampMin method, which was characterized by high performance in other publications [12,14]. As representatives of the local-similarity group, the kNN, LLS, LSA, and RF methods were selected, each of which was indicated as the most effective in individual works [6,12,13,14,15,16,17,18,19,20]. Among the global-similarity methods, two methods representing different approaches to the principal component analysis (BPCA and PPCA) were selected, as well as the SVD method, which is not based on PCA analysis and uses matrix dimension reduction.

Furthermore, this study examines the influence of key additional factors that may affect imputation performance, including the choice of protein identification algorithm and the application of logarithmization to the data as a preprocessing step. By addressing these aspects, the study provides a comprehensive framework for assessing the robustness and utility of imputation techniques in proteomic analyses, contributing to the advancement of reliable quantitative methodologies in the field.

## 2. Results

Protein identification and the handling of missing values are fundamental components in ensuring the reliability of quantitative proteomic analyses. In this study, we evaluated the performance of two widely used protein identification tools, FragPipe and MaxQuant, and assessed the efficacy of various imputation methods applied to the resulting datasets. This section presents the results of the comparative evaluation, highlighting the differences in protein identification between FragPipe and MaxQuant, as well as the corresponding imputation times.

### 2.1. Protein Identification

FragPipe and MaxQuant identified 3508 and 4987 proteins, respectively. This result is 42% better for MaxQuant, but the identification time was almost 10 times faster for FragPipe (1693 min. for MaxQuant, 178 min. for FragPipe). However, the programs achieved very similar results both in terms of the share of missing values in the full dataset (ca. 51%) and the share of proteins containing all values correctly identified (ca. 27%).

### 2.2. Imputation Time

The slowest imputation method is clearly the RF method, although it is worth noting that the method works faster with an increase in the MV share. The time differences between the other methods are negligible; only the kNN and BPCA methods require a few seconds more than the rest.

### 2.3. NRMSE

**Figure 1:**
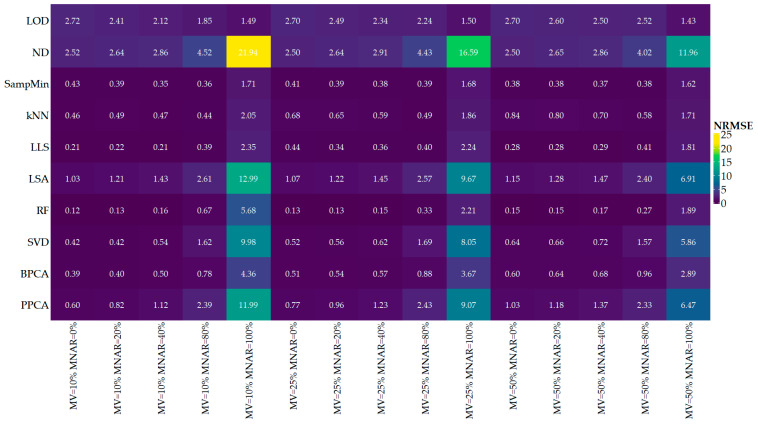
Mean NRMSE values for the data obtained using the FragPipe program in the form of a heatmap. The rows correspond to the tested imputation methods, and the columns to the subsequent combinations of the total MV content and the share of MNAR values.

The mean NRMSE values obtained for individual methods for FragPipe data and presented in Figure 1 may be interpreted as the absolute imputation error because they are calculated as the difference between the imputed value and the true value. Lower NRMSE values indicate a lower imputation error.

For a better understanding of the behavior of individual methods, it is interesting to consider the extreme cases of the share of MNAR values, i.e., 0% MNAR and 100% MNAR. However, these are artificial cases; they do not apply to real data, which are a mixture of MNAR and MAR values. They specify the nature of individual imputation methods, but they should be interpreted separately from the rest of the cases. With a zero share of MNAR values, the best result in terms of imputation error expressed by NRMSE is obtained by the RF method, followed by LLS and kNN. The worst results are obtained by the LOD and ND methods, which is in line with expectations for left-censored methods. Global methods in these conditions obtain worse results with the increase in the MV share, while for the other methods it does not matter. At a 100% MNAR value share, the best results are achieved by the left-censored methods, i.e., LOD and SampMin, and the local-similarity methods: kNN and LLS. Again, the global-similarity methods improve with the increase in MV, an effect that is also observed for the ND, LLS, LSA, and RF.

With the increase in the MV share, the imputation error increases for the LOD, SampMin, and BPCA methods, and decreases only for the RF method. For the kNN and LSA methods, it has a negligible effect. At a low share of the MNAR values for the SVD and PPCA methods, the imputation error initially increases, but when their share increases to 80%, we begin to observe the opposite trend. The increase in the share of MNAR values has a negative impact on the ND, LLS, LSA, and global-similarity methods, with the most significant effect observed for the LSA method. The opposite behavior is observed for the single-value methods LOD and SampMin. No significant impact is observed for the kNN and RF methods.

**Figure 2:**
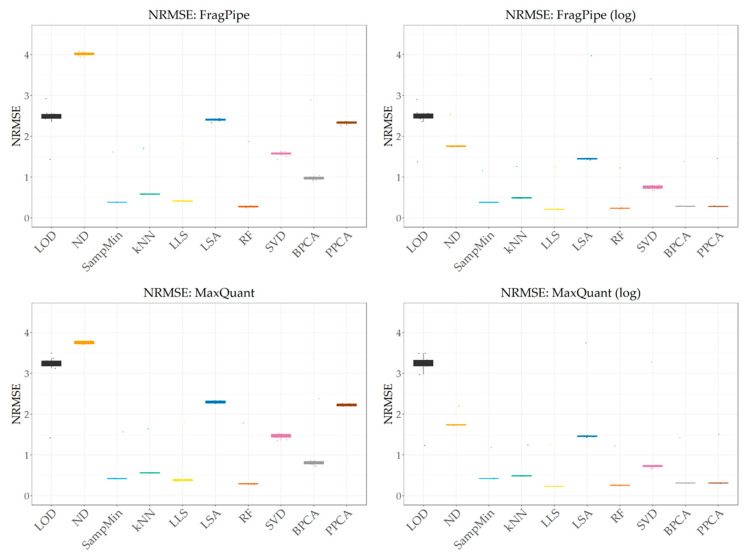
NRMSE values obtained for both identification programs before and after logarithmization at 50% MV and 80% MNAR share.

Figure 2 shows the case that best reflects the conditions of the real experiment, i.e., MV = 50% and a high share of MNAR values = 80% for both identification programs, before and after logarithmization. The behavior of the individual imputation methods for data obtained using both programs with respect to increasing the share of MV and MNAR is the same. The imputation error for data from FragPipe and MaxQuant is comparable. In the case of FragPipe, the obtained NRMSE values are slightly better only for the LOD method. For MaxQuant, however, better results are obtained for the ND and global-similarity methods, which is probably due to more data being available.

Carrying out the logarithmization process before imputation does not negatively affect the imputation error for any of the methods. For the LOD, SampMin, kNN, and RF methods, no significant effect is observed, while for the remaining methods (ND, LLS, LSA), the results are visibly improved, especially for the global-similarity methods (SVD, BPCA, PPCA), which achieve some of the best results in such conditions.

### 2.4. ROC Curve

**Figure 3:**
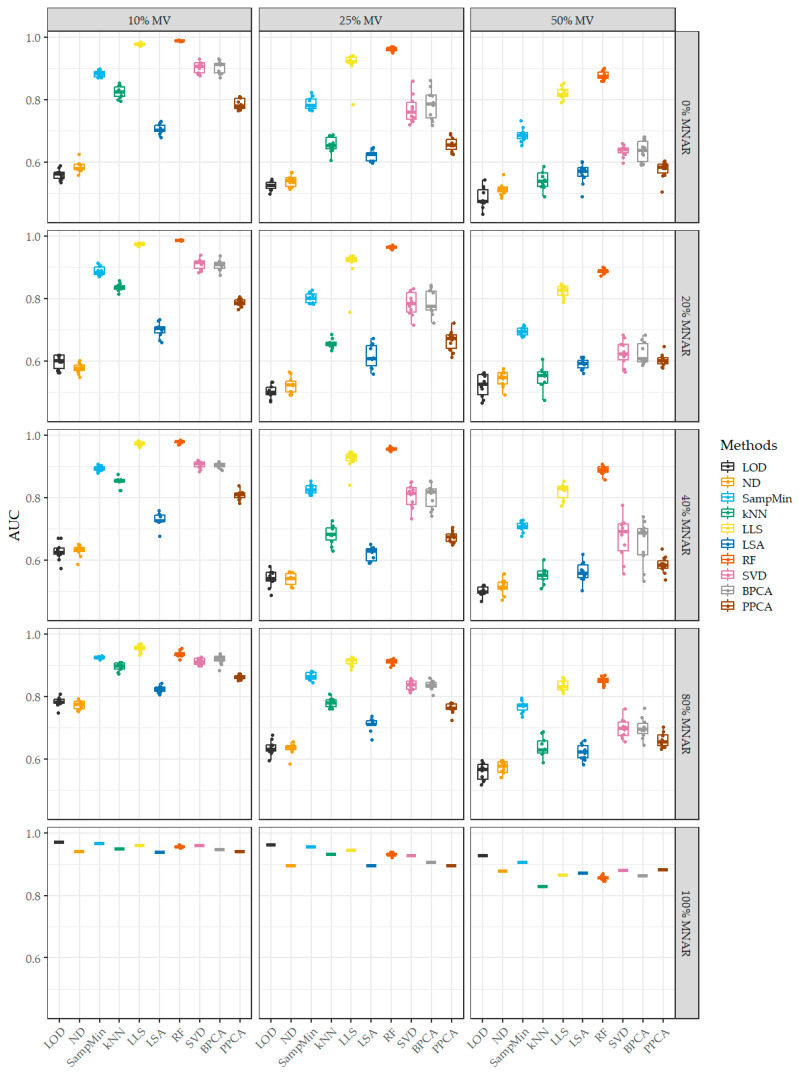
The obtained AUC values for the data obtained using the FragPipe program in the form of box plots. Each box corresponds to a subsequent combination of the total MV content and the share of MNAR values.

The ROC curve shows the relationship between the true-positive rate (TPR) and the false-positive rate (FPR) at different decision thresholds. It allows you to visualize the effects of changing the threshold (as an adjusted *p*-value) and allows you to choose it in such a way as to balance the need to minimize FPR with the need to maximize TPR. Another use of the ROC curve is to compare the performance of classification models, which, in the context of this experiment, means a measure of how well a given imputation method performs in reproducing the actual results. The area under the ROC curve (AUC) was used to compare the classification of proteins as true or false positives with respect to the actual data, which, as a single-number parameter, is easier to compare and visualize. An AUC value of 1 indicates excellent classification ability of the model, while a value of 0.5 indicates no discriminatory ability, comparable to random guessing. Figure 3 shows all the AUC values obtained for the FragPipe data, similarly to NRMSE. With a 0% share of MNAR values, the best result, similarly to NRMSE, was obtained by the RF and LLS methods. One of the single-value methods—SampMin—also obtained a high result for these conditions. All the methods achieve the highest results with all combinations of MV and MNAR when the share of MNAR values is 100%. The observed scatter of results is also significantly reduced for this condition.

An increase in the number of missing values has a negative impact on all the tested imputation methods. However, with the increase in the share of MNAR values, the classification accuracy increases significantly for the LOD and ND methods. The SampMin, kNN, LSA, and PPCA methods also improve but to a lesser extent. The increase in the MNAR value share negatively affects only the LLS and RF methods. The RF and LLS methods achieve slightly better results, and the kNN method improves in the case of a low share of MNAR values. In the case of the LSA method, and especially the global methods, a clear improvement is visible, but in critical conditions, i.e., with a 100% share of the MNAR values, they perform worse than before logarithmization.

**Figure 4:**
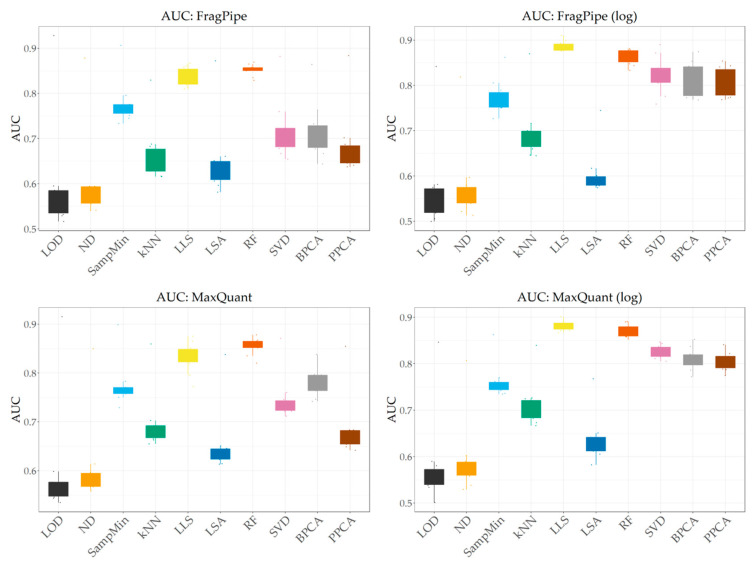
AUC values obtained for both identification programs before and after logarithmization at 50% MV and 80% MNAR share.

Comparing the results for the FragPipe and MaxQuant data for real-experiment conditions in Figure 4, the results obtained by the individual imputation methods are again very similar for both identification programs. In this case, kNN, SVD, and BPCA achieve slightly better AUC values for MaxQuant data, and for the other methods the differences are negligible.

As in the case of NRMSE, prior logarithmization has a positive effect on the obtained results in most cases. Noticeably better AUC values can be observed for the kNN, LLS, and global-similarity methods. The greatest improvement due to logarithmization can be observed for the PPCA method. For the ND method, logarithmization resulted in worse results, contrary to NRMSE. The other methods behave the same regardless of the logarithmization conditions.

### 2.5. MV Imputation on Full Dataset

In the second stage of the imputation method evaluation, the full datasets with naturally occurring MVs were used and the data were then filtered, retaining only those proteins that are present in at least 50% of the samples, which resulted in 1709 proteins for FragPipe and 2404 proteins for MaxQuant. This represented 48.7% and 48.2% of the total number of proteins initially identified, respectively.

**Figure 5:**
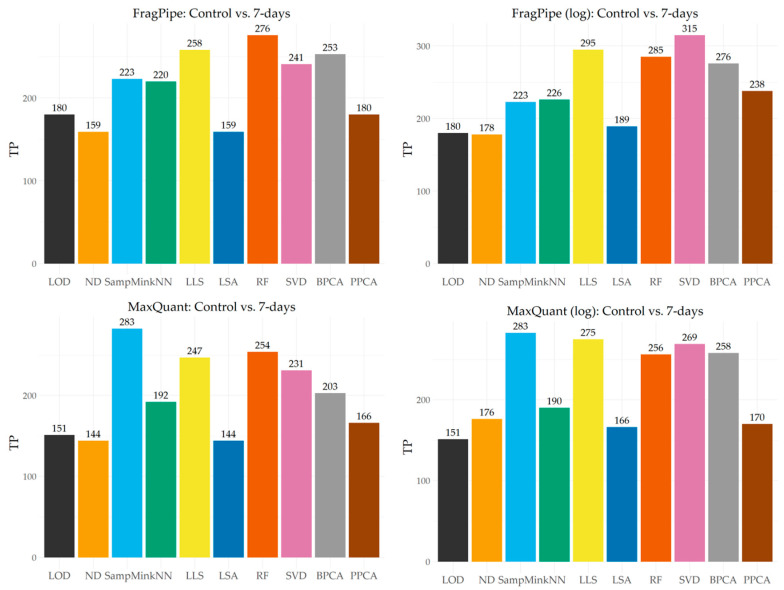
TP values obtained for both identification programs before and after logarithmization.

After data imputation, the t-Student test (FDR = 5%) was performed, and the adjusted *p*-value and fold change were calculated. Figure 5 shows the number of proteins identified as true positives by each imputation method for FragPipe and MaxQuant data before and after logarithmization. Without logarithmization, the highest number of proteins identified as true positives was observed for the RF, LLS, and BPCA methods in the case of FragPipe and for the SampMin, RF, and LLS methods in the case of MaxQuant. The worst results were obtained for the ND and LSA methods for both protein identification programs. The vast majority of the imputation methods achieved a better result for FragPipe despite a smaller total number of identified proteins. The largest differences in performance were observed for the LOD (ca. 20%) and BPCA (ca. 25%) methods, and the smallest for the LLS method (ca. 4%). For the MaxQuant program, only the SampMin method achieved a better result but by as much as 27%. Data logarithmization did not improve the results achieved only for the LOD and SampMin methods, and worsened the results only for the kNN method in the case of MaxQuant. For the other methods, the number of proteins identified as true positives increased, but the percentage scale of improvement differed depending on the identification program used. In this respect, the largest difference was noted for the PPCA method, which improved by as much as 32% for FragPipe and by only 2% for MaxQuant. Among the imputation methods that improved, the smallest differences can be observed for the RF method. Previous logarithmization once again had a very positive effect on global imputation methods, but in this test this tendency was more pronounced for the FragPipe program and it was the SVD method, next to LLS and RF, that achieved the best results under these conditions. For the MaxQuant program, the largest increase in TP value was noted for another global imputation method—BPCA–, but the best result was still achieved by the SampMin method.

Including FC in the considerations, Volcano plots were drawn and the number of statistically significant proteins at FC = 2 and FDR = 5% was compared. The obtained results differ, sometimes significantly, depending on the logarithmization or its absence and the program used to identify proteins. Unfortunately, in this case there is no absolute reference point on the basis of which it is possible to state which imputation method gives better, more reliable results. Any overexpression in the case of this experiment can be expected to occur on the side of the 7- and 21-day groups, but this is not unambiguous. A larger number of statistically significant points also does not necessarily indicate better performance of a given imputation method, because despite performing a number of statistical tests, some overestimations may still occur, leading to later overinterpretations. For the FragPipe program, significantly more high points can be observed, which correspond to proteins with very low FDR. For the LOD method, overexpression occurs on the control group side for the FragPipe program, and for the MaxQuant program on the 7-day group side, and logarithmization of data does not affect the number of statistically significant points and their distribution in any way. This is in agreement with the results for TP similarly to the SampMin method, with the difference that overexpression for the 7-day group is observed here regardless of the program used. For the ND method, logarithmization causes a clear equalization of the number of significant points on both sides and an increase in their total number, and the results for both identification programs are relatively similar. The graphs for the methods that achieved the best results for tests on data with artificially implemented MV, i.e., RF and LLS, are quite similar. Logarithmization of data does not affect the number of statistically significant points and overexpression in this case, which occurs on the 7-day group side, although their total number is greater for the FragPipe program. In the case of the remaining methods, i.e., kNN, LSA, SVD, BPCA, and PPCA, there is a decrease in the number of statistically significant points for imputation performed after data normalization. This effect is greater for the MaxQuant program in the case of the LSA, SVD, and BPCA methods and especially for the PPCA method. In each of the mentioned cases, overexpression occurs on the side of the 7-day group both before and after logarithmization. For the mentioned methods, more statistically significant points are observed for the FragPipe program. The largest number of statistically significant points can be noted for the PPCA method for the FragPipe program before logarithmization.

For the obtained imputation results, PCA analysis was also performed using only statistically significant proteins (FDR = 5% and FC = 2). Complete separation of the studied groups before and after logarithmization was observed for the LOD, ND, and LSA methods. For the PPCA method, complete separation was also noted before logarithmization, but for the logarithmized datasets, only partial separation of the groups occurred and this is the only imputation method for which a negative trend was observed. In the case of imputation methods for which the separation of the studied groups was minimal or only partial, after logarithmization the separation of groups increases for the SampMin, RF, SVD, and BPCA methods. No clear effect of logarithmization on the separation of the studied groups was noted for the kNN and LLS methods.

## 3. Discussion

The evaluation of extreme cases involving missing values, specifically with 0% and 100% of missing not at random (MNAR) values, was instrumental in understanding how individual imputation methods perform under conditions where missing data are either left-censored or completely random. These conditions allowed for an in-depth examination of the methods’ sensitivity to different types of missing data patterns, contributing valuable insights into their strengths and limitations. As expected, and consistent with findings from previous studies [6,10,11,12,13,14], single-value imputation methods, LOD, and SampMin demonstrated strong performance in scenarios with a high proportion of missing not at random (MNAR) values. These methods are particularly effective when the missing data exhibit systematic patterns or are subject to censoring, where imputation is required to approximate the true values. Even the ND method, despite exhibiting a higher imputation error (as measured by NRMSE) under these conditions, showed comparable classification efficiency (AUC) to the other methods. This highlights the nuanced nature of the ND method, which, while less precise in imputation, can still perform well in maintaining the integrity of the overall dataset classification. When all missing values were random (0% MNAR), none of the imputation methods showed significant improvements in performance, reinforcing the challenge of handling completely random missing values. However, in line with previous findings [10,14], the local-similarity methods, especially RF and LLS, emerged as the most effective approaches under these conditions, yielding the best results in terms of imputation accuracy and classification performance. This suggests that local-similarity methods are better equipped to deal with the inherent randomness of missing data, as they leverage the relationships and patterns within the available data to impute missing values more effectively. The SampMin method, which has previously been highlighted as one of the best imputation methods [12,14], also performed remarkably well in this context, further supporting its utility in dealing with missing data, particularly in cases where missing values exhibit a non-random pattern. Overall, most of the methods that have been identified as top performers in earlier studies—SampMin [12,14], kNN [15,16,17,18], RF [6,11,18,19,20], and BPCA [21]—consistently delivered high-quality results, both in terms of imputation error and the identification of true positives. Among the methods that have not been very popular so far, but which achieved very high results in a given experiment, we can mention the LLS and SVD after log-normalization. Limitations of the methods described, depending on the case, may include imputation time, the need for optimization, or difficulty of implementation. The slowest process is definitely the RF method; it is counted in minutes and even took 30 times longer than the imputation time of the second-in-line method, kNN, proportional to the size of the data. An undoubted advantage is the lack of necessity of optimization, in contrast to kNN and LLS (k-nearest neighbors) and global-similarity methods (nPCs). Implementation of the above methods is slightly more difficult than in the case of single-value methods, which are often built into the software for processing proteomic data and require certain, at least minimal programming skills. Differences in the performance of individual methods were observed, which can largely be attributed to the experimental conditions, such as the choice of protein identification program (FragPipe vs. MaxQuant), as well as the impact of data preprocessing steps like logarithmization. The comparison of the results before and after logarithmization confirms the statement [3], according to which normalization should be performed immediately before data imputation, and some methods, especially global-similarity methods such as SVD [9], are particularly sensitive to this factor. These factors influenced the final imputation outcomes and highlighted the importance of optimizing both the choice of imputation method and the preprocessing pipeline to suit the specific characteristics of the dataset. The findings underscore that while there is no one-size-fits-all solution for imputation [12], certain methods, particularly local-similarity approaches, exhibit robust performance across various missing value scenarios, offering valuable tools for improving data quality in proteomics. The results of this study have significant implications for the reliability and accuracy of proteomic analyses, particularly in the context of large-scale quantitative studies where missing data are inevitable. The choice of imputation method can influence the overall dataset quality, and, thus, the interpretation of protein expression profiles, which are critical for downstream analysis and conclusions. By identifying the strengths and limitations of various imputation techniques, this study provides valuable guidance for optimizing proteomic workflows, ensuring that missing data do not compromise the validity of the findings. The improved performance of local-similarity methods like LLS and RF, particularly under random missing data conditions, highlights their potential for enhancing the robustness of proteomic datasets, which are often subject to high variability and incomplete data due to experimental constraints. In the context of medical diagnostics, these findings could have a profound impact on the interpretation of biomarker discovery and disease diagnostics. Proteomic data are increasingly used to identify disease-related biomarkers, monitor disease progression, and assess therapeutic efficacy. The ability to accurately impute missing values ensures that proteomic datasets remain consistent and reliable, even in the presence of incomplete data, which is common in clinical settings. For example, in diagnosing complex diseases, such as cancer or neurodegenerative disorders, where protein expression profiles play a key role, reliable imputation methods could improve the identification of disease-specific biomarkers and facilitate the development of personalized therapeutic strategies. The ability to accurately address missing values in proteomic data not only improves the quality of scientific analyses but also enhances the clinical applicability of proteomics. The results obtained for the volcano plots and PCA analysis show that the wrong choice of imputation method can lead to false conclusions about the biological functionality of selected proteins. For example, the use of methods such as LOD and ND in subsequent statistical tests led to obtaining a large number of statistically significant proteins and a good separation of the studied groups for these points. Considering the results from the first part of the experiment, where both methods showed very low imputation accuracy, it can be expected that such results result from the underestimation of certain values and, consequently, the overestimation of changes in expression. For studies on the border of medicine and biology, this could lead to indicating incorrect biomarkers or indicating incorrect factors influencing the development of the disease.

Furthermore, the importance of assuring data validity in proteomics cannot be overstated, as it directly influences the reliability of diagnostic results. A recent publication [20] on a standardized protocol for assuring the validity of proteomics results from liquid chromatography–high-resolution mass spectrometry outlines essential steps to ensure result integrity. This protocol, based on the ISO/IEC 17025:2017 [21] and ISO 15189:2022 [22] standards, provides a comprehensive approach to quality control in proteomic research, further emphasizing the need for stringent validation processes in mass spectrometry-based proteomics. From a metrological perspective, the evaluation of imputation methods ensures data quality and comparability across studies, which is essential for clinical diagnostics. Logarithmization and careful imputation can minimize biases and improve the reproducibility and accuracy of proteomic measurements, supporting more reliable diagnostic tools. These findings suggest that optimizing imputation strategies is key for advancing proteomics in both research and clinical settings. Future studies should focus on evaluating these methods across additional datasets and at the peptide level, further refining the utility of proteomics in precision medicine. By integrating robust quality control protocols, such as those outlined in recent standards, proteomic analyses can meet the high standards required for diagnostic applications, advancing the field of precision medicine.

## 4. Materials and Methods

### 4.1. Samples

In this study, the most effective imputation methods identified in individual publications were curated and applied to data derived from a real experimental setting. The dataset originated from a study involving rat brain samples, analyzed in the context of fluorine-containing drug administration. A comprehensive description of the experimental results and the analytical procedures employed in this investigation has been detailed in our prior paper [23].

A proteomics label-free approach was used to prepare and analyze the samples. Firstly, sample preparation was performed, which included protein extraction, reduction, alkylation, and in-solution digestion with tripsin. In the second step, chromatographic separation by liquid chromatography (nano-UHPLC, Thermo Scientific, Bartlesville, OK, USA) and analysis by high-resolution mass spectrometry (MS; Orbitrap; Thermo Scientific, Bartlesville, OK, USA) were used to obtain the data. The experimental workflow for this proteomics approach was also described previously [24].

### 4.2. Chemicals, Reagents, and Instrumentation

Analytical grade chemicals and analytical standards were obtained from Merck (Darmstadt, Germany), Promega (Madison, WI, USA), Thermo Scientific (Bartlesville, OK, USA), and EMD Millipore (Darmstadt, Germany). Deionized water from the Milli-Q system (18.2 MΩ cm; EMD Millipore, Darmstadt, Germany) was used for samples and standard dilution. The instrumentation for extraction and sample preparation was as follows: mechanical homogenizer Ultra-Turrax (IKA, Königswinter, Germany), laboratory incubator CLN 240 (MultiSerw, Brzeźnica, Poland), vacuum centrifuge 5804/5804 R (Eppendorf, Enfield, CT, USA), vortex shaker (IKA, Königswinter, Germany), thermomixer Eppendorf Comfort (Eppendorf, Enfield, CT, USA), and vacuum concentrator SpeedVac Concentrator Plus (Eppendorf, Enfield, CT, USA). Reversed-phase capillary nano-UHPLC separations were performed using an UltiMate 3000 nano system (Dionex Ultimate Series UHPLC, Thermo Scientific, Bartlesville, OK, USA) equipped with in-house–packed capillary C-18 column (75 µm × 500 mm, particle size 1.9 µm) coupled on-line with a high-resolution tandem mass spectrometer (Orbitrap Fusion Tribrid™ Mass Spectrometer, Thermo Scientific, Bartlesville, OK, USA).

### 4.3. Protein Identification

The recorded MS/MS spectra were used to identify the proteins using two different algorithms to compare their possible effects on the performance of the imputation methods. For this purpose, the FragPipe (v. 17.1) and MaxQuant (v. 2.0.3.0) programs were used using the MSFragger [25] and Andromeda algorithms [26], respectively. The SwissProt database (accessed on 25 March 2022) was searched, with the specified taxonomy Rattus Norvegicus (Taxonomy ID: 10116). Proteins and peptides were identified using a target–decoy approach with a reversed database. As the fixed modification, propionamidation on cysteine (C) was set, which derived from using acrylamide as an alkylating agent. RAW files were used to identify proteins using the MaxQuant program. The mass tolerance was set to ±10.0 ppm for parent ion masses and ±0.6 Da for fragment ion masses. The false discovery rate for PSM and peptides was set at 1%. To perform identification using FragPipe, it was necessary to convert the raw data to the *.mzXML format with the Mass Converter (v. 1.0.3) R package [27]. The precursor and fragment ion mass tolerance was set to ±10.0 ppm and a maximum isotope error option (0/1/2/3) was used. Only the MaxLFQ intensities [28] calculated by both identification programs were used to test the imputation methods, which allowed for an optimal comparison of the obtained results. Additionally, to check the influence of the logarithmization process, an evaluation of the imputation methods was performed before and after the data logarithmization.

### 4.4. Imputation Methods

The LOD, ND SampMin, and LSA algorithms were developed and implemented in-house. The LLS, SVD, BPCA, and PPCA methods were taken from the R package library *pcaMethods* [29] (accessed on 10 June 2024). The kNN and RF algorithms were derived from the *VIM* [30] (accessed on 10 June 2024) and *missForest* [31] libraries (accessed on 10 June 2024), respectively. To test the individual imputation methods and analyze the results, a script in R (v. 4.4.0) was used.

For the ND method, the mean value (µ) and standard deviation (σ) were assumed as µ_i_ = µ_m_ − 2.2σ_m_ and σ_i_ = 0.3σ_m_ (where i = values for imputation, m = measured values) based on previous work [32]. For the kNN and LLS methods, the number of nearest neighbors k, also understood as the number of similar proteins, was 6 and 150, respectively. For the RF method, standard parameters (ntree = 100) were used. For the global-similarity methods, the number of principal components (nPCs) was optimized and was set to 4 for the SVD and BPCA, and nPCs = 1 for the PPCA.

**Figure 6:**
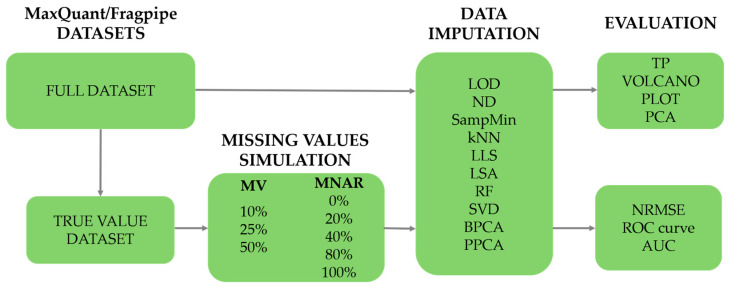
Scheme of imputation method evaluation.

The study of imputation methods consisted of two stages and the procedure is presented in Figure 6. In the first, only those proteins for which all the values were correctly determined were listed from the obtained dataset. Missing values were implemented with variously defined ratios of the total MV and MNAR using the algorithm presented by Jin et al. [6]. The MV ratios were 10%, 25%, and 50% and the MNAR ratios were 0%, 20%, 40%, 80%, and 100%, for a total of 15 combinations. The chosen scope of the MV ratios makes it possible to examine the behavior of individual imputation methods with a low, medium, and high share of missing values and to observe the trends with increasing MV. Extreme values of MNAR (0% and 100%) were used to verify the thesis about the effectiveness of left-censored methods for non-random missing values [7] and to indicate the group of methods that performs best in the opposite case. The remaining MNAR ratios (20%, 40%, 80%), similarly to MV, were used to observe the behavior of imputation methods with an increasing share of MNAR, i.e., whether there are certain maxima for imputation accuracy or whether the trend is increasing or decreasing. The case that best reflects the conditions of a real experiment is the ratio MV = 50% [3] and MNAR = 80% [7], i.e., when about half of the data consist of missing values and missing not at random values dominate in this set.

Each combination was replicated 10 times, resulting in a total of 150 test data sets. In order to evaluate the performance of the imputation methods by comparing the imputed values with real data, normalized root mean square error (NRMSE) implemented from the *missForest* [31] R package was used (accessed on 10 June 2024). NRMSE introduces normalization, making the result independent of the data scale, which facilitates the comparison of different models operating on different datasets and has been used to assess the imputation error in other works [6,33,34]. Raw datasets before MV implementation were used to classify proteins as TP or FP using an adjusted *p*-value at 5%. Then, this classification was performed on each of the imputed datasets and the classification results were compared, which allowed for the plotting of the ROC curve and the calculation of the area under the curve (AUC). In the second stage, imputation by selected methods was carried out on the full datasets, and the evaluation of the imputation methods was based on the number of true-positive results, fold change (FC), and PCA.

## 5. Conclusions

This study highlights the robustness and versatility of random forest and local least-squares imputation methods, which achieved the highest performance in terms of normalized root mean square error, area under the ROC curve, and the number of proteins accurately classified as true positives. Other methods, including SampMin, k-nearest neighbors, singular value decomposition, and Bayesian principal component analysis, also delivered commendable results, though their efficacy was often context-dependent. These findings underscore the importance of selecting imputation methods tailored to specific dataset characteristics and experimental conditions. Logarithmization emerged as a critical preprocessing step, particularly for global-similarity methods like singular value decomposition, improving data consistency and interpretability without negatively impacting imputation performance. Its consistent utility suggests that log-normalization should be a standard practice before data imputation in proteomics workflows. The study also noted algorithm-specific variability, such as the substantial differences observed in SampMin performance between datasets processed with MaxQuant and FragPipe, emphasizing the need to optimize imputation strategies for specific workflows. From a metrological perspective, the study highlights the critical role of robust imputation methods in ensuring data quality, reliability, and comparability across proteomic studies. Proteomic datasets are inherently prone to variability due to experimental constraints, and missing data further complicates result consistency. Methods like random forest and local least-squares minimize biases and maintain the integrity of quantitative analyses, which is vital for both research and clinical applications. Aligning imputation strategies with quality control standards, such as ISO/IEC 17025:2017 and ISO 15189:2022, enhances the reproducibility and reliability of protein quantification. Logarithmization further supports this goal by stabilizing variance and reducing biases, ensuring consistency in data interpretation. The integration of imputation methods with validated metrological frameworks is crucial for advancing biomarker discovery and diagnostic applications, particularly in clinical settings where data integrity directly impacts outcomes. These findings emphasize the role of optimized imputation strategies in achieving high standards of measurement reliability, fostering the development of robust proteomic workflows. Future research should explore the application of these methods across diverse datasets, including peptide-level analyses, while further integrating metrological frameworks to ensure data quality and comparability. This will advance the use of proteomics in precision medicine and diagnostics, ensuring robust and reliable analytical outcomes.

## Data Availability

The authors confirm that the data supporting the findings of this study are available within the article. Raw data that support the findings of this study are available from the first author, upon reasonable request.
